# Evaluation of Radiofrequency Ablation Safety and Efficacy in Primary Hyperparathyroidism: A Single‐Center Retrospective Study in Taiwan and Literature Review

**DOI:** 10.1002/kjm2.70022

**Published:** 2025-05-19

**Authors:** Shu‐Ting Wu, Wei‐Che Lin, Chih‐Ying Lee, Cheng‐Kang Wang, An‐Ni Lin, Yen‐Hsiang Chang, Shun‐Yu Chi, Chen‐Kai Chou

**Affiliations:** ^1^ Division of Endocrinology and Metabolism, Department of Internal Medicine Kaohsiung Chang Gung Memorial Hospital and Chang Gung University College of Medicine Kaohsiung Taiwan; ^2^ Department of Diagnostic Radiology Kaohsiung Chang Gung Memorial Hospital and Chang Gung University College of Medicine Kaohsiung Taiwan; ^3^ Department of Nuclear Medicine Kaohsiung Chang Gung Memorial Hospital and Chang Gung University College of Medicine Kaohsiung Taiwan; ^4^ Department of Surgery Kaohsiung Chang Gung Memorial Hospital and Chang Gung University College of Medicine Kaohsiung Taiwan

**Keywords:** parathyroid adenoma, primary hyperparathyroidism, radiofrequency ablation

## Abstract

Radiofrequency ablation (RFA) is increasingly recognized as a minimally invasive option for primary hyperparathyroidism (PHPT). This study aimed to evaluate the effectiveness and safety of ultrasound (US)‐guided RFA for the treatment of PHPT in Taiwan. We conducted a retrospective study of patients with PHPT who underwent RFA at a single medical center between March 2020 and January 2023. Serum biochemical samples, changes in parathyroid nodule volume, symptomatic scores, and complications were analyzed at follow‐up after RFA. The volume reduction ratio (VRR) and treatment response at 1 year were evaluated. Among the 31 patients, 93.55% achieved a complete response with a VRR of 97.48% at 12 months post‐RFA. Serum calcium and intact parathyroid hormone (iPTH) levels were significantly lower immediately after treatment than at baseline. However, a difference in phosphorus levels was noted 1 week later, and the alkaline phosphatase (ALP) level was significantly decreased after 3 months (*p* < 0.05). Transient hoarseness occurred in three patients and resolved spontaneously within 6 months. US‐guided RFA is effective and safe for treating PHPT, with a satisfactory VRR and treatment response. This approach could be an alternative to surgery for ineligible patients.

## 1 Introduction

Primary hyperparathyroidism (PHPT) is a prevalent endocrine disorder characterized by hypercalcemia with elevated or inappropriately normal plasma parathyroid hormone levels [1, 2]. The primary etiology of PHPT is a solitary parathyroid adenoma, which accounts for approximately 80% of all PHPT cases [2]. This condition commonly manifests after the age of 50 years and has a higher incidence in women. Owning to the widespread use of biochemical testing, PHPT is now frequently diagnosed earlier and can be asymptomatic. The classic symptoms of “stones, bones, and groans” associated with PHPT are now considered rare. Parathyroidectomy remains the standard treatment for PHPT and is typically recommended for patients with symptomatic disease [3, 4]. Parathyroidectomy for PHPT has a success rate exceeding 95% when performed by experienced surgical teams [5, 6]. Although minimally invasive parathyroidectomy techniques are associated with a low surgical risk [7], there are situations in which patients may decline surgery, or their clinical conditions may render them unsuitable for surgery.

In recent decades, advances in thermal ablation techniques have revolutionized the management of various tumors. Radiofrequency ablation (RFA) has emerged as a minimally invasive alternative for treating PHPT, particularly beneficial for patients with advanced age or significant comorbidities, as it reduces the risks associated with general anesthesia [8, 9, 10]. Typically performed on an outpatient basis under local anesthesia [11], RFA has demonstrated high success rates in achieving postoperative normocalcemia and alleviating PHPT‐related symptoms [9, 10, 12, 13]. Specifically, RFA achieves a 92% volume reduction rate (VRR) [12]. A systemic literature review has shown that there is currently no definitive standard guideline for the thermal ablation of parathyroid adenomas, and management decisions are primarily based on evidence from small case series. This study aimed to evaluate the efficacy and safety of ultrasound (US)‐guided RFA for the treatment of PHPT.

## 2 Methods

### 2.1 Participants

From March 1, 2020 to January 31, 2023, 31 patients with biochemically confirmed PHPT from the outpatient department were enrolled in this study. Patients were eligible if they met the following criteria: (1) serum intact parathyroid hormone (iPTH) levels exceeding the upper limit of the normal range (88 pg/mL); (2) positive technetium 99 m‐sestamibi single‐photon‐emission‐computed tomography (CT)/CT (sestamibi SPECT/CT) or conventional CT results; (3) detectable hyperplastic parathyroid glands on US examination; (4) aspiration biopsy confirming benign nodule with an elevated parathyroid hormone washout concentration; and (5) either refusal or ineligibility for surgery. Patients with parathyroid lesions exhibiting suspicious malignant sonographic features (such as heterogeneity of contents, lobulated morphology, or local invasion) or those with fine‐needle aspiration‐confirmed parathyroid carcinomas were excluded. Additionally, individuals with secondary or tertiary hyperparathyroidism, a history of thyroid or parathyroid surgeries, symptoms of hoarseness, or any vocal cord motility abnormalities were not included in the study cohort. This retrospective study was approved by the Chang Gung Medical Foundation Institutional Review Board (IRB No. 202301450B0), and written informed consent was obtained from all patients for both the US‐guided fine‐needle aspiration and RFA procedures prior to their implementation.

### 2.2 Data Collection and RFA Procedure

The baseline characteristics collected included age, sex, and serum levels of iPTH, calcium, phosphorus, alkaline phosphatase (ALP), and creatinine. Symptom scores related to hyperparathyroidism were recorded using a questionnaire assessing symptoms, such as ostealgia, arthralgia, pruritus, calcinosis cutis, and restless legs. Neck US and sestamibi single‐photon emission CT scans were used to determine the size and location of the parathyroid gland before the procedure. Three orthogonal diameters of the parathyroid glands (the largest diameter and two perpendicular diameters) were measured using sonography. The volume of the parathyroid glands was calculated using the formula: *V* = *πabc*/6 (*V*: volume; *a*: the largest diameter; *b* and *c*: the other two perpendicular diameters).

**FIGURE 1:**
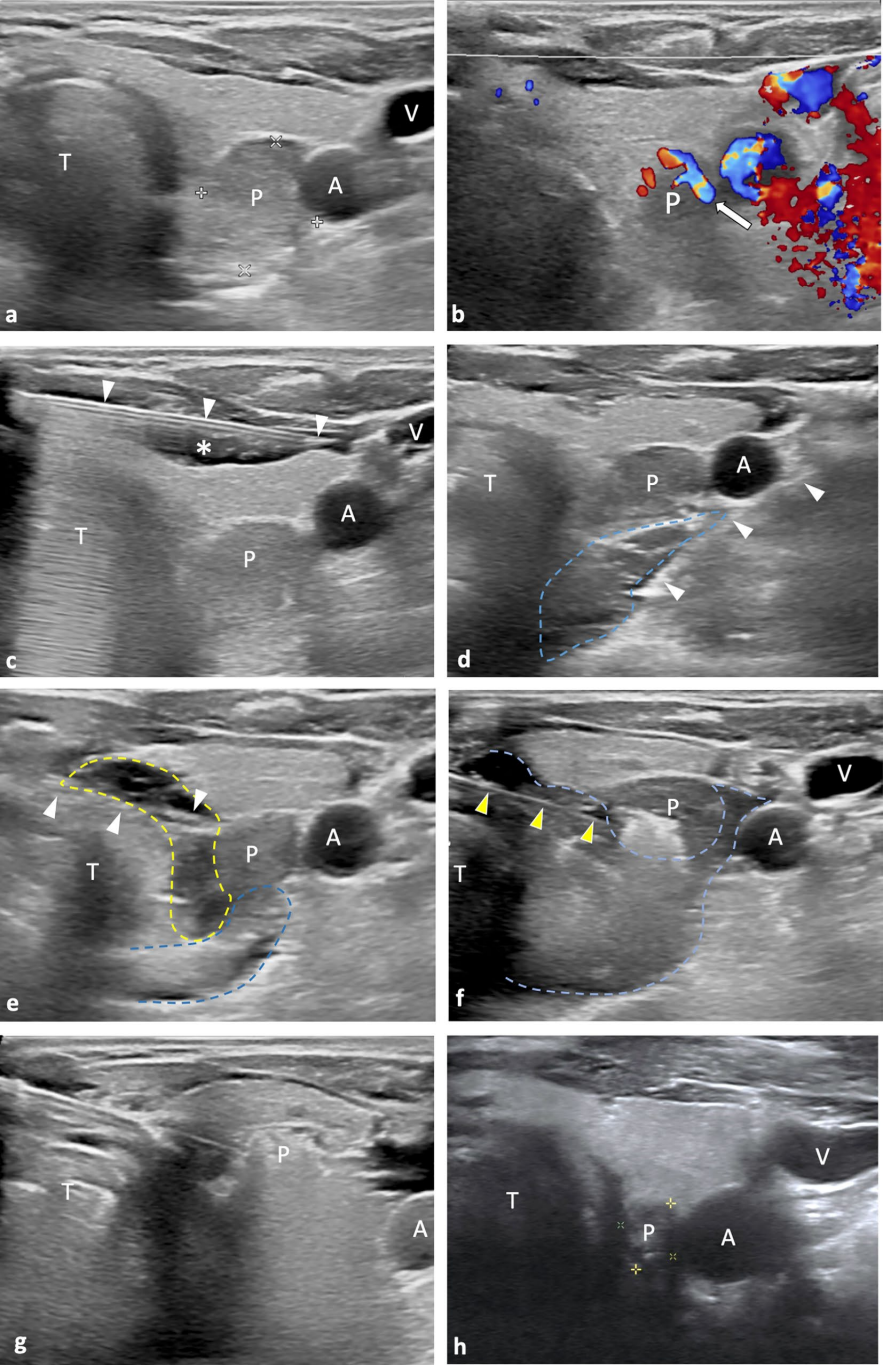
An 85‐year‐old male with a right lower parathyroid adenoma underwent RFA. (a) Preoperative ultrasound revealed a well‐circumscribed homogenously hypoechoic, ovoid parathyroid adenoma (measuring 1.4 × 1.6 × 2.2 cm) located at the lower pole of the right lobe of thyroid gland. (b) Color Doppler imaging shows a characteristic extrathyroidal feeding vessel (arrowhead) entering the parathyroid adenoma. Internal vascularity exhibits a typical peripheral distribution. (c) Local anesthetic was injected via a needle (arrowhead) above the thyroid capsule using a 2% lidocaine hydrochloride solution (star). (d) For lateral approaching hydrodissection, the 5% glucose solution was continuously injected via a needle (arrowhead) at the thyroid bed (blue dashed line) to prevent thermal damage. (e) For medial approaching hydrodissection, the 5% glucose solution was continuously injected via a needle (arrowhead) between tracheal and parathyroid adenoma (yellow dashed line). (f) After positioning the tip of RFA electrode needle (yellow arrowhead) in the deepest area of the parathyroid adenoma, sequential ablation was performed by moving the tip back and forth, from deep to superficial in each plane. (g) The procedure was considered complete when the parathyroid adenoma changed to transient hyperechogenicity. (h) At the one‐year follow‐up, a significant volume reduction of right lower parathyroid adenoma was observed, leaving a residual scar (0.8 × 0.8 × 0.6 cm, VRR = 92.2%). A, carotid artery; P, parathyroid adenoma; T, tracheal; V, internal jugular vein; VRR, volume reduction ratio.

RFA was performed on an outpatient basis by a radiologist (W.C.L.) with over a decade of experience in US‐guided procedures. All patients underwent a single RFA session. After local anesthesia was administered to the subcutaneous and thyroid capsules using a 2% lidocaine dilution, hydrodissection was performed using 5% dextrose water (D5W). This technique created a protective buffer between the adenoma and surrounding critical structures, reducing thermal injury risks and allowing for more aggressive ablation. Specific attention was paid to the critical triangle, carotid space, trachea, and esophagus during the procedure. The moving‐shot technique was used to ablate the parathyroid adenoma slice by slice under real‐time sonographic guidance. RFA was performed with power settings ranging from 20 to 30 W, with adjustments made based on lesion characteristics to optimize thermal ablation while minimizing tissue damage. The entire RFA procedure took approximately 15–20 min. Complete ablation of the parathyroid adenoma was assessed in real time based on hyperechoic changes observed on US imaging. An example of a patient with a right parathyroid adenoma who underwent RFA is shown in Figure 1. After the procedure, the patients were instructed to apply pressure to their necks for 30 min and then apply ice packs to reduce tissue edema and local bleeding.

### 2.3 Follow‐Up Evaluation

Follow‐up evaluations were conducted at 1 h, 1 week, and 1, 3, 6, and 12 months post‐RFA. Neck US and serum biochemical samples, including iPTH, total calcium, phosphorus, and ALP levels, were obtained during each follow‐up period. Complete response was defined as the restoration of normal serum calcium and iPTH levels at the 12‐month follow‐up. A partial response was defined as a reduction in serum iPTH and/or calcium levels that remained above the upper limit of the normal range after RFA. The VRR was calculated using the formula: VRR (%) = [(initial volume—final volume) × 100]/initial volume. Complications and symptomatic improvements were assessed during the follow‐up.

The safety of the treatment was evaluated by collecting all reported complications and adverse effects during the follow‐up. The assessed complications included hoarseness, hypocalcemia, subcutaneous edema, skin burns, hematoma, infection, and any life‐threatening events occurring during or after RFA.

### 2.4 Statistical Analysis

Data analyses were performed using IBM SPSS Statistics Version 25. Continuous variables were presented as mean ± standard deviation or as median and interquartile range (IQR). Wilcoxon's signed‐rank test was used to compare changes in the VRR with the previous follow‐up and changes in the biochemical profile before RFA and at each follow‐up. All statistical tests were two‐sided, and *p* < 0.05 was considered statistically significant.

## 3 Results

Of the 33 patients initially identified 31 were included in the final analysis. Two patients were not excluded because of missing imaging studies or laboratory tests. The patient characteristics and treatment responses are summarized in Table 1. Among the 31 patients, 22 (71.0%) were women, with a mean age of 61.0 ± 13.5 years. Twenty patients were asymptomatic, whereas the remaining patients reported fatigue (9.7%), nephrolithiasis (12.9%), ostealgia (35.4%), and osteoporosis (48.4%). The median diameter and volume of the parathyroid nodules were 1.7 cm (IQR: 1.3–2.2 cm) and 0.69 cm^3^ (IQR: 0.36–1.51 cm^3^), respectively. Twenty of the parathyroid nodules were observed on the inferior side.

**TABLE 1:** Demographic and clinical characteristics of patients (*n* = 31).

Clinical characteristic	All patients
Sex (*n*)	
Female	22 (71.0%)
Male	9 (29.0%)
Mean age at diagnosis (year)	61.0 ± 13.5
Symptomatology	
Asymptomatic (*n*)	20 (64.5%)
Fatigue (*n*)	3 (9.7%)
Nephrolithiasis (*n*)	4 (12.9%)
Ostealgia (*n*)	11 (35.4%)
Osteoporosis (*n*)	15 (48.4%)
Nodule volume (cm^3^)	0.69 (0.36–1.51)
Maximum nodule diameter (cm)	1.7 (1.3–2.2)
Nodule location	
Upper right	6 (19.4%)
Upper left	5 (16.1%)
Lower right	10 (32.3%)
Lower left	10 (32.3%)
Serum iPTH (pg/mL)	164.3 (121.5–288.1)
Serum calcium (mg/dL)	11.41 ± 1.01
Serum phosphorous (mg/dL)	2.85 ± 1.15
Serum alkaline phosphatase (U/L)	91.65 ± 68.39
Complete response	29 (93.5%)
Partial response	2 (6.5%)

*Note:* Non‐normally distributed continuous variables were presented as median (interquartile range). Categorical variables were presented as *n* (%).

Abbreviation: iPTH: intact parathyroid hormone.

Serum hormone and biochemical laboratory examinations were conducted before RFA treatment, with a median serum iPTH value of 164.3 pg/mL (IQR: 121.5–288.1 pg/mL). The mean serum calcium and phosphorus levels were 11.41 ± 1.01 mg/dL and 2.85 ± 1.15 mg/dL, respectively. The mean serum ALP level was 91.65 ± 68.39 U/L.

**FIGURE 2:**
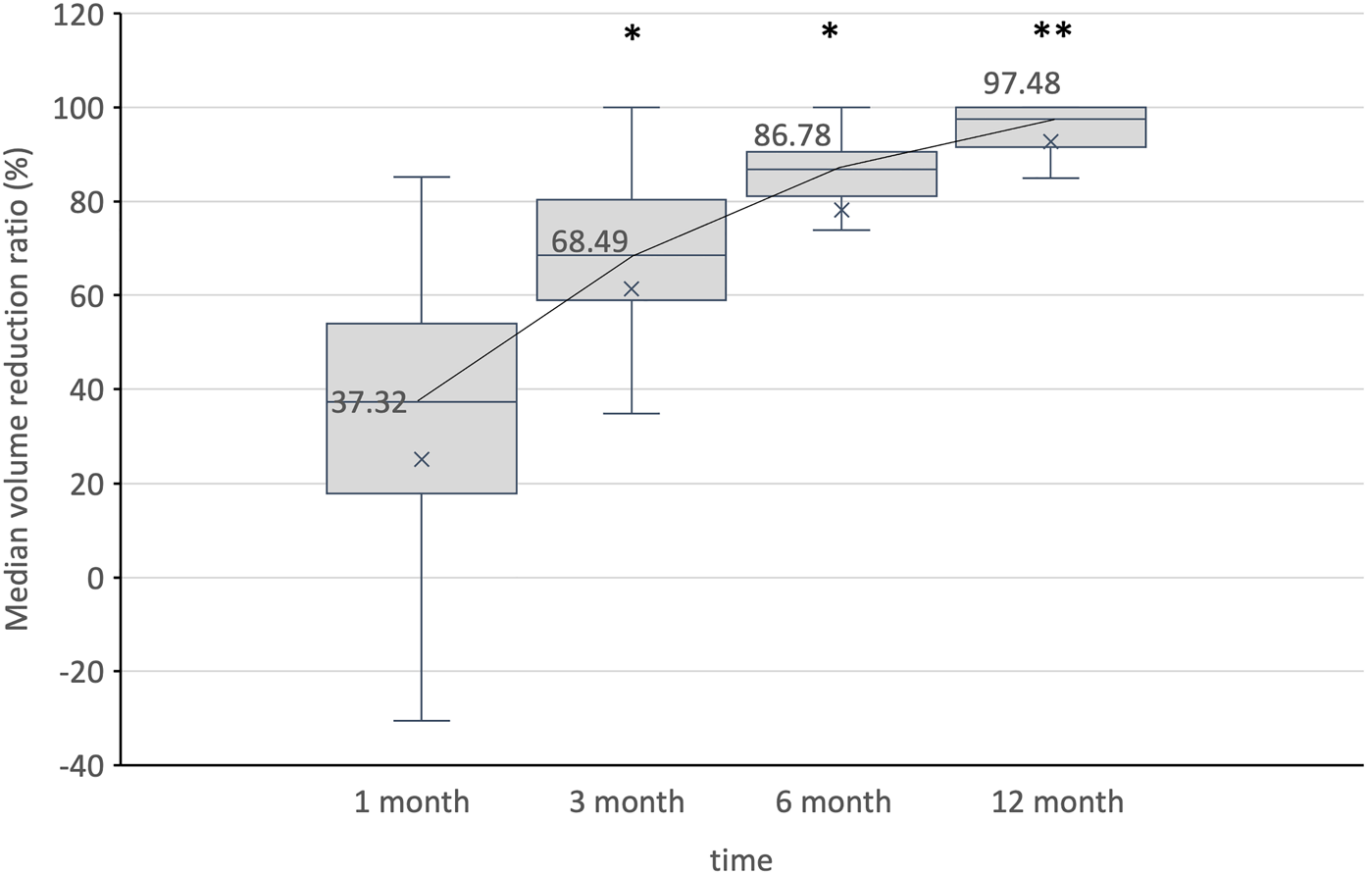
The boxplot with trend line shows the median volume reduction ratio (VRR) in patients treated with ultrasound‐guided radiofrequency ablation during the study period. *p < 0.001 versus previous follow‐up VRR. **p < 0.05 versus previous follow‐up VRR.

**FIGURE 3:**
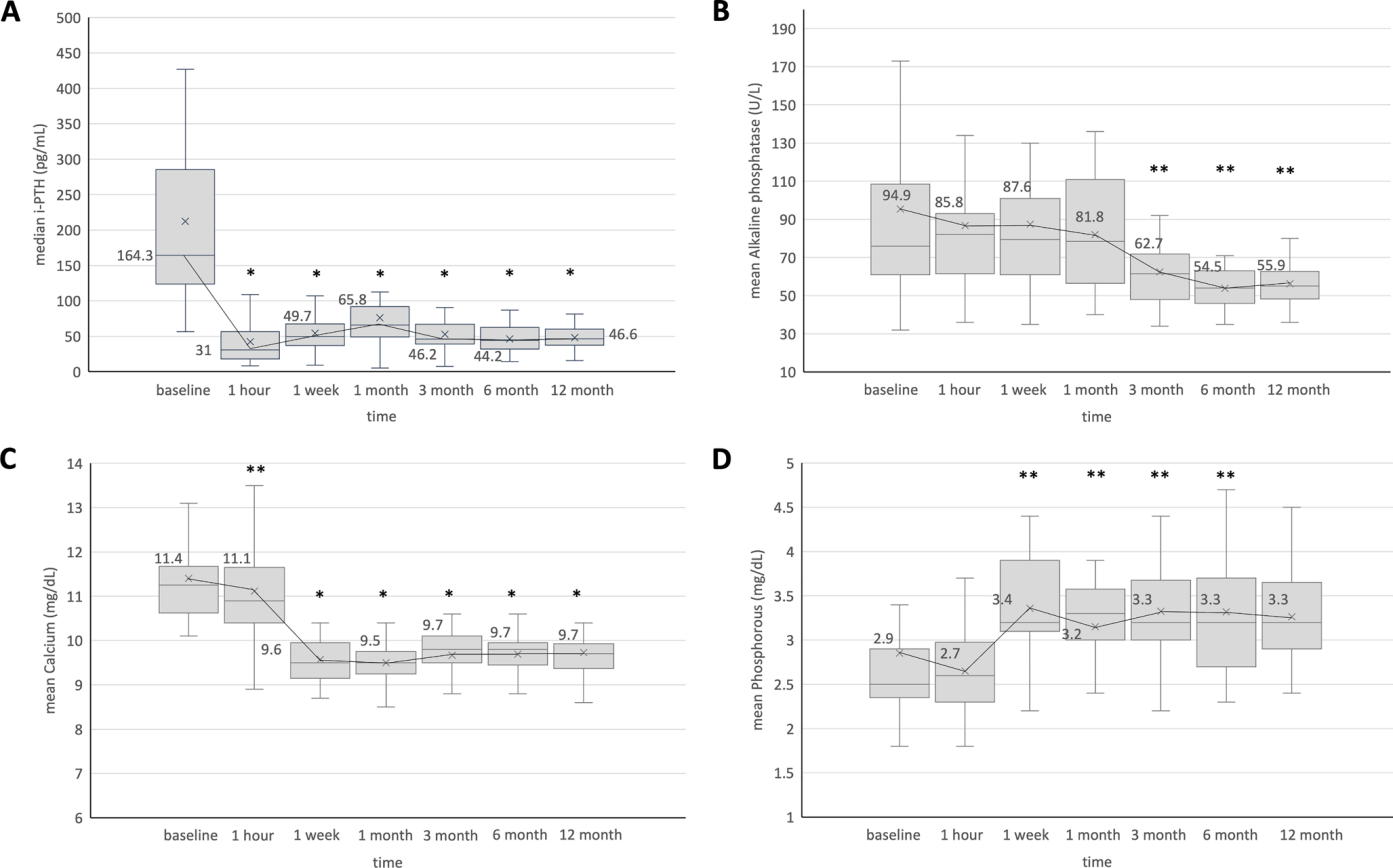
Serum i‐PTH level (A), Alkaline phosphatase (B), Calcium (C), Phosphorous (D) in patients treated with ultrasound‐guided radiofrequency ablation during the study period. *p < 0.001 versus before ablation. **p < 0.05 versus before ablation. i‐PTH, intact parathyroid hormone.

All patients underwent neck US evaluations at 1, 3, 6, and 12 months after the RFA procedure, followed by annual evaluations. The median VRR showed significant changes over a follow‐up period of up to 12 months (Figure 2). At 12 months post‐RFA, the median VRR was 97.48%. All 31 patients experienced a decrease in serum calcium and iPTH levels after RFA, with 29 demonstrating a complete response, characterized by the normalization of both serum calcium and iPTH levels. Figure 3 shows the changes in these parameters during the follow‐up period after RFA. Significant differences were observed immediately after the intervention in iPTH and calcium levels. However, a difference in phosphorus levels was noted 1 week later, and the ALP level significantly decreased after 3 months (*p* < 0.05).

## 4 Discussion

In this study, we demonstrated that RFA is an effective, safe, and feasible treatment for patients with parathyroid adenomas. After RFA, we observed a significant decrease in serum iPTH and calcium levels. Additionally, there was a notable reduction in the size of the lesions, as assessed by using neck ultrasonography.

Surgical parathyroidectomy remains the standard treatment for parathyroid adenomas causing PHPT [2]. Bilateral neck exploration (BNE) has traditionally been the surgical approach for PHPT, with cure rates ranging from 95% to 98% when performed by experienced surgeons, and with low complication rates [5]. Minimally invasive parathyroidectomy has emerged as the preferred approach to BNE because of its safety and efficacy [7]. Minimally invasive parathyroidectomy can be performed under local or general anesthesia and has been shown to have comparable success rates to BNE at 6 months, with a low incidence of perioperative hypocalcemia (14.4% vs. 26.5%; *p* = 0.02) [14]. RFA is a minimally invasive technique for patients who are unfit for parathyroid adenoma surgery. In our cohort, we achieved an excellent complete response rate of 93.5% after RFA for parathyroid adenomas, which is comparable to outcomes reported in previous studies (ranging from 48.3% to 97.4%) (Table 2). A study comparing the clinical outcomes of RFA and parathyroidectomy for PHPT showed no significant differences in biochemical cure rates at 12 months (RFA vs. parathyroidectomy: 80.0% vs. 83.9%; *p* = 0.684) [19]. These findings suggest that RFA can be a cost‐effective, nonsurgical treatment option for PHPT, with safety and efficacy comparable to those of parathyroidectomy.

**TABLE 2:** Literature search of noncase report studies analyzing RFA treatment for PHPT.

Authors (year), country	Case, *n*	Hypercalcemic PHPT, *n*	Adenoma size (range), cm	Complete response, *n* (%)	Partial response, *n*	Complications, (*n*)	Follow‐up duration, month
Sormaz et al. (2017), Turkey [9]	5	5	2.8 (1.1–6.2)	3 (60.0%)	2	Hypocalcemia (1)	~6
Korkusuz et al. (2018), Germany [10]	9	8	NR	5 (55.6%)	4	0	~3
Ha et al. (2020), South Korea [12]	11	11	2.0 (0.6–3.8)	7 (63.6%)	4	Transient hypocalcemia (1)	13.6 ± 18.7
Khandelwal et al. (2020), India [13]	8	8	1.4 (0.9–3.5)	6 (75.0%)	0	transient hoarseness (1)	18
Ebrahiminik et al. (2022), Irlan [15]	27	27	NR, (volume: 0.58 cm^3^)	NR	NR	0	12
Peng et al. (2022), China [16]	51	51	NR, (volume: 0.63 cm^3^)	44 (86.3%)	1	Transient hoarseness (3), hypocalcemia (8)	12
Chai et al. (2022), China [17]	39	34	1.6 cm	38 (97.4%)	1	Transient hoarseness (2), transient hypocalcemia (7)	13.2 ± 4.6
Utrero et al. (2023), Spain [18]	29	29	1.2 cm	14 (48.3%)	11	Transient hoarseness (1), persisted hoarseness (1)	16.3 ± 7.2
Current study, Taiwan	31	31	1.7 (0.9–3.5)	29 (93.5%)	2	Transient hoarseness (3)	12

Abbreviations: NR, not reported; PHPT, primary hyperparathyroidism; RFA, radiofrequency ablation.

Microwave ablation (MWA) is another thermal ablation technique that has shown advantages in the treatment of PHPT [20]. Both MWA and RFA have been recommended for the treatment of PHPT, offering key advantages such as minimal invasiveness, high tolerance, and repeatability. Few studies have compared the outcomes of RFA and MWA in PHPT. A prospective cohort study from China showed no difference in cure rates between the MWA and RFA groups for PHPT (80.22% vs. 80.49%; *p* = 0.971) [21], which is consistent with a previous multicenter study [22]. A meta‐analysis comparing the efficacy and safety of MWA and RFA in patients with PHPT and refractory secondary hyperparathyroidism demonstrated that while MWA had a shorter operation time for a single lesion and a higher complete ablation rate for larger lesions than those of RFA, there was no significant difference in the cure rate or complications of PHPT between MWA and RFA (*p* > 0.05) [23]. The cure rate of thermal ablation is slightly lower than that of surgical resection [24, 25]. Patients who underwent MWA had significantly less blood loss and shorter surgical times than those of patients who underwent surgical resection (*p* < 0.001) [25]. However, owing to the limited number of studies, high‐quality reliable evidence is still needed to determine whether RFA is superior to MWA for treating PHPT.

**FIGURE 4:**
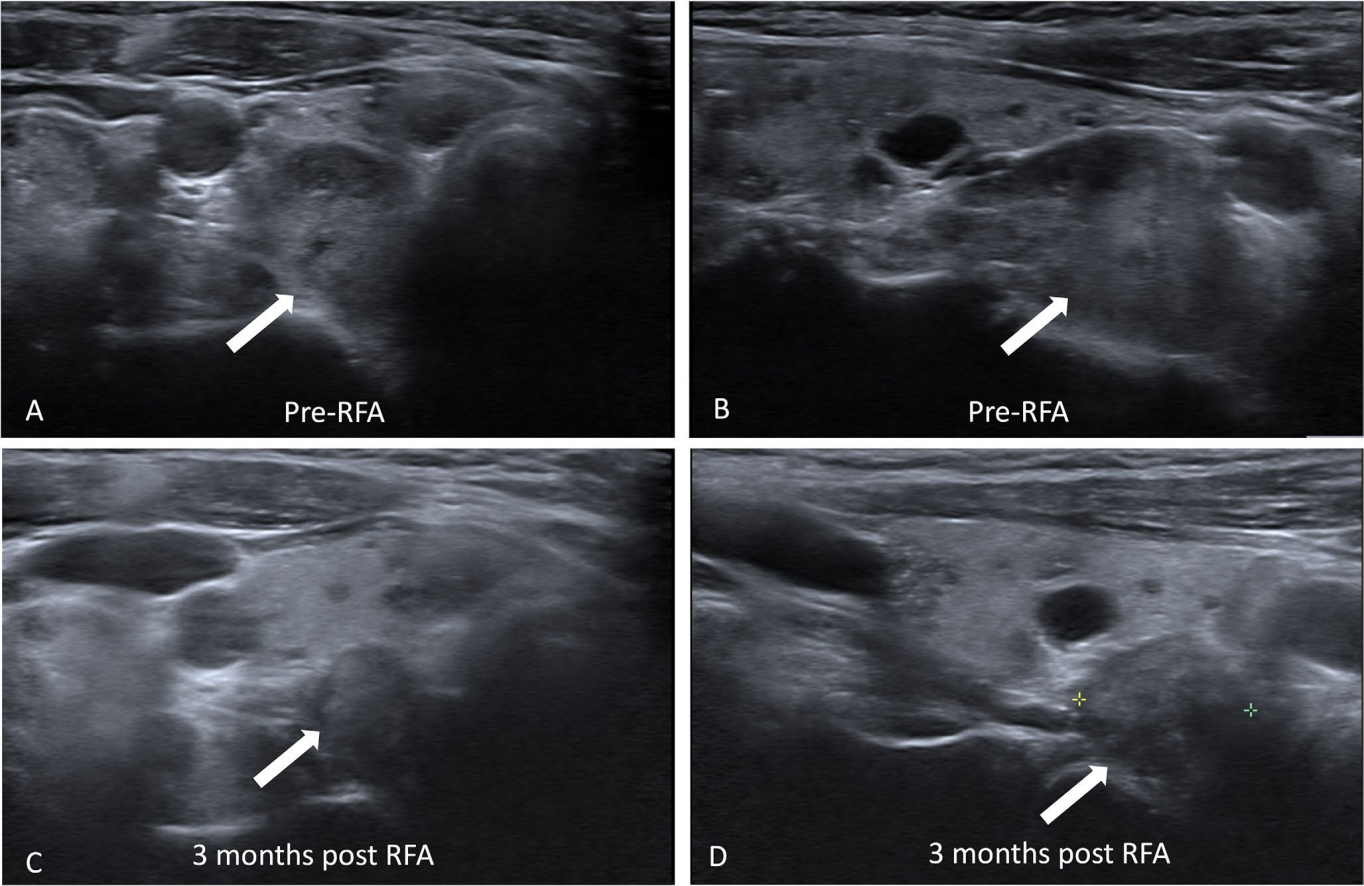
A 66‐year‐old female with primary hyperparathyroidism. (A, B). Pre‐RFA ultrasound examination revealed a right parathyroid adenoma (1.8 x 1.3 x 3.0 cm) (white arrow). (C, D). After RFA, the volume of the nodule (0.9 x 1.1 x 1.4 cm) (white arrow) gradually decreased at the 3‐month follow‐up.

In this study, the rate of complete response after RFA at 12 months was 93.5%. Only two patients with a partial response developed elevated iPTH levels after 1 and 6 months, respectively, while serum calcium levels remained within the normal range. This phenomenon is defined as eucalcemic parathyroid hormone elevation (ePTH). A 66‐year‐old woman with a relatively large right parathyroid adenoma (3.0 cm) demonstrated a gradual decrease in nodule volume on follow‐up US after RFA (Figure 4). Another 85‐year‐old man with a larger nodule (2.7 cm) and higher preoperative iPTH level (730.1 pg/mL) could not undergo complete treatment because of the nodule size and limited cooperation during the RFA procedure.

The incidence of ePTH after parathyroidectomy has been reported to range from 8% to 40% [26, 27, 28]. Thermal ablation also leads to the development of ePTH. The partial response observed in these two patients might be associated with the size of the nodule and preoperative levels of iPTH. Previous studies have identified several risk factors for ePTH, including older age, lower 25‐hydroxyvitamin D3 levels, higher preoperative iPTH levels, and elevated serum creatinine levels [16, 26, 28, 29]. Consistent with previous surgical cohort analyses, ePTH after RFA is a dynamic and transient phenomenon that does not predispose patients to recurrent PHPT [27, 30].

Transient hoarseness was observed in three patients (9.7%) after RFA, and all recovered spontaneously within 6 months after RFA. No other complications, such as hypocalcemia or hungry bone syndrome, were noted after the procedure. Consistent with previous studies, our study demonstrated that transient hoarseness is a common adverse effect, with an incidence ranging from 5.1% to 12.5%, according to recently published studies (Table 2). A possible explanation for complications after RFA is thermal injury to the recurrent laryngeal nerve or vagus nerve during the ablation process, or a transient blockage by lidocaine injection. Therefore, the hydrodissection technique, which involves the injection of a 5% dextrose solution between the nerve and tumor, is crucial for preventing thermal damage to the nerve [12, 31]. However, few studies have compared the safety of thermal ablation with that of parathyroidectomy for PHPT. Liu et al. [25] demonstrated that the occurrence of complications was similar between thermal ablation and parathyroidectomy, whereas patients undergoing thermal ablation experienced less blood loss and shorter surgical times. Considering the absence of serious and permanent complications observed in our study, it is reasonable to consider RFA a safe therapeutic option for PHPT.

To the best of our knowledge, the present study is the first in Taiwan to assess the efficacy of RFA in the treatment of PHPT in patients without previous parathyroidectomy. Our study has several limitations. First, this was a retrospective study with a relatively small sample size, which may have increased selection bias. Second, this study only examined the short‐term effect of RFA; therefore, further studies should focus on long‐term outcomes. Third, this study focused solely on the efficacy and safety of RFA for PHPT and did not include a direct comparison between RFA and parathyroidectomy. Prospective comparative studies are needed to evaluate their relative efficacy, safety, and long‐term outcomes.

In conclusion, RFA is a safe and effective treatment for patients with PHPT. It can serve as an alternative treatment modality for patients who cannot tolerate general anesthesia or surgical resection.

## Conflicts of Interest

The authors declare no conflicts of interest.

## Data Availability

The data that support the findings of this study are available on request from the corresponding author. The data are not publicly available due to privacy or ethical restrictions.
